# Generalization of Wei's urn design to unequal allocations in sequential clinical trials

**DOI:** 10.1016/j.conctc.2015.12.007

**Published:** 2016-01-14

**Authors:** Wenle Zhao, Viswanathan Ramakrishnan

**Affiliations:** Department of Public Health Sciences, Medical University of South Carolina, Charleston, SC 29425, USA

**Keywords:** urn design, Unequal allocation, Randomization

## Abstract

Wei's urn design was proposed in 1987 for subject randomization in trials comparing *m* ≥ 2 treatments with equal allocation. In this manuscript, two modified versions of Wei's urn design are presented to accommodate unequal allocations. First one uses a provisional allocation of $r_{1}^{2}:r_{2}^{2}$ to achieve the target allocation*r*_1_:*r*_2_, and the second one uses equal allocation for *r*_1_ + *r*_2_ arms to achieve an unequal allocation*r*_1_:*r*_2_ based on the concept Kaiser presented in his recent paper. The properties of these two designs are evaluated based on treatment imbalance and allocation predictability under different sample sizes and unequal allocation ratios. Simulations are performed to compare the two designs to other designs used for unequal allocations, include the complete randomization, permuted block randomization, block urn design, maximal procedure, and the mass weighted urn design.

## 1 Introduction

Based on the generalized Friedman's urn model [1], Wei proposed an urn design for sequential trials comparing *m* ≥ 2 treatments with equal allocations in 1978, as a compromise between the complete randomization (CR), which may result in large treatment imbalances, and the permuted block randomization (PBR), which has high allocation predictability [2]. Wei's urn design, denoted byUD(*w,α,β*), starts from an urn with*w* balls color coded for each of the*m* ≥ 2 treatments. When a subject is ready for randomization, a ball is drawn and replaced. The subject is assigned to the treatment represented by the ball. Then α more balls for the treatment and *β* more balls for each of the other treatments are added to the urn. Wei proved that the unconditional allocation probability for each treatment assignment in the UD converges to the equal allocation. Wei also indicated that the treatment allocation predictability of the UD was lower than that of the PBR, and the treatment imbalance was comparable to that of Pocock and Simon's Minimization method [2], [3]. The UD is easy to implement and is considered as one of the commonly used restricted randomization method in clinical trials [4].

In recent years, the use of unequal allocation in clinical trials is growing, partially due to the emergence of Bayesian adaptive designs [5] and response adaptive randomization [6] motivated by ethical, trial efficiency, economical, and patient recruitment feasibility considerations [4]. However, randomization designs for unequal allocations are largely limited to CR and PBR. The generalizing of the UD to unequal allocations has received some attentions. For example, in their book published in 2002, Rosenberger and Lachin briefly described a procedure to generalize Wei's UD from equal allocation to two-arm unequal allocations [4]. Recently in 2012, Kaiser pointed out that this generalization is incorrect and provided a fix for a specific scenario of unequal allocation 2:1 [7]. In the same article, Kaiser described another randomization strategy for unequal allocation*r*_1_:*r*_2_ between the experimental and the control arms as to perform randomization for equal allocation to*r*_1_ + *r*_2_ treatment arms, and then combine*r*_1_ of these arms for the experimental arm assignment and*r*_2_ of these arms for control [7]. Kaiser did not provide details on the statistical properties of these two unequal allocation randomization procedures. In this manuscript, these two procedures described by Kaiser for unequal allocations are rigorously defined, evaluated, and compared to other commonly used unequal allocation randomization methods. Evaluation criteria include the unconditional allocation probability, the allocation predictability, the treatment imbalance, and their advantages and limitations under different trial scenarios. In Section 2, notations and measures used in this article are defined. In Section 3, a modified version of Wei's UD is proposed by using a provisional allocation. In Section 4, an alternate approach using Kaiser's equal allocation randomization is introduced. In Section 5, the performances of these two designs are compared with other randomization methods, and in Section 6 a discussion is provided.

## 2 Notations and measures

Let *n* be the sample size, *m* be the number of treatment arms, *n*_*ij*_ denote the number of subjects assigned to treatment*j* after*i* subjects have been randomized in to the study, and *b*_*ij*_ represent the number of balls in the urn for treatment*j* after*i* subjects randomized. Let *p*_*ij*_ be the conditional allocation probability of assigning subject*i* to treatment*j*. In an urn model, there is$p_{ij}=b_{i-1,j}/\sum_{k=1}^{m}b_{i-1,k}.$ Let *u*_*ij*_ be the unconditional allocation probability of assigning the *i*th subject to treatment*j* prior to the start of the trial. To prevent selection bias, it is desired that the unconditional allocation probability equals the target allocation probability for each treatment assignment [7], [10].

Let $r_{1}^{*}:r_{2}^{*}:\cdots :r_{m}^{*}$ represent the allocation ratio desired by the study design. For example, when two treatments are compared to one single control, 1:1:2 is the optimal allocation defined by Dunnett [9]. By default, an allocation ratio is expressed in terms of allocation probabilities with the sum of allocation elements equals to 1. For example, 1:1:2 = 0.2929:0.2929:0.4142. Let *r*_1_:*r*_2_:⋯:*r*_*m*_ be the allocation ratio targeted by the randomization algorithm. For example, using PBR for the Dunnett allocation, one may choose 2:2:3 = 0.2857:0.2857:0.4286 or 5:5:7 = 0.2941:0.2941:0.4118 as the target allocation. Recognizing the difference between the desired allocation and the target allocation is important because not all randomization designs are able to target any desired allocation. A randomization design is valid only if it has an asymptotic allocation equal to the target allocation. Based on the notations described above, the following measures are defined for the evaluation of randomization designs:

*Allocation precision* is measured by the Euclidian distance between the achieved allocation and the target allocation after*i* subjects randomized, $d_{i}=\sqrt{\sum_{j=1}^{m}{\left(n_{ij}-ir_{j}\right)}^{2}}$.

*Allocation accuracy* is measured by the Euclidian distance between the target allocation and the desired allocation multiplied by the sample size [8], $\eta =\sqrt{\sum_{j=1}^{m}{\left(r_{j}-r_{j}^{*}\right)}^{2}}$.

*Allocation error*$d_{i}^{*}$ is measured by the Euclidian distance between the achieved allocation and the desired allocation after*i* subjects. It increases as *i* increases when*η* is not negligible.

$$d_{i}^{*}=\sqrt{\sum_{j=1}^{m}{\left(n_{ij}-ir_{j}^{*}\right)}^{2}}=\sqrt{d_{i}^{2}+2i\sum_{j=1}^{m}\left(n_{ij}-ir_{j}\right)\left(r_{j}-r_{j}^{*}\right)+i^{2}\eta^{2}}$$

*Allocation predictability* is defined by the Euclidian distance between the conditional allocation probability and the target allocation probability for each treatment assignment [8], $\varphi_{i}=\sqrt{\sum_{j=1}^{m}{\left(p_{ij}-r_{j}\right)}^{2}}.$

*φ*_*i*_ equals zero when the CR is applied. Unlike to the correct guess probability defined based on the Blackwell and Hodges' convergence strategy [11], which applies to equal allocations only, measure (4) generally applies to both equal and unequal allocations.

Desired features for a good randomization design for unequal allocations include:

a) High allocation accuracy, represented by a small value of*η*, ideally*η*=0.
b) High allocation precision, represented by a small value in$\bar{d}=\sum_{i=1}^{n}d_{i}/n$.
c) Low allocation predictability, represented by a small value in$\bar{\varphi }=\sum_{i=1}^{n}\varphi_{i}/n$.
d) Unconditional allocation probability equals to, or at least converges to, the target allocation probability, i.e.*p*_*ij*_ = *r*_*j*_ for *i* = 1,2,⋯; and*j* = 1,2,⋯,*m*.

## 3 A modified urn design with provisional allocation

### 3.1 An unequal allocation urn procedure needs to be modified

Wei's UD(*w,α,β*) for *m* ≥ 2 equal allocations can be defined by the conditional allocation probability$p_{ij}\left(UD\right)=\frac{w+\alpha n_{i-1,j}+\beta \left(i-1-n_{i-1,j}\right)}{wm+\alpha \left(i-1\right)+\beta \left(i-1\right)\left(m-1\right)}$ for *i* = 1,2,⋯;*j* = 1,2,⋯,*m* [12]. Historically, only integers are used for the constants*w,α*, and *β* in the UD for easy illustration purpose. Theoretically,*w* and *β* can be any positive number, *α* can be any real number. The ratios *α*/*w* and *β*/*w* determine the UD. Let*w* = 1, Wei's UD procedure can be specified by$p_{ij}\left(UD\right)=\frac{1+\alpha n_{i-1,j}+\beta \left(i-1-n_{i-1,j}\right)}{m+\alpha \left(i-1\right)+\beta \left(i-1\right)\left(m-1\right)}$. Rosenberger and Lachin described a modified urn design (mUD) for two-arm trials targeting an unequal allocation of*v*_1_:*v*_2_ [4]. Their urn starts from Refs. *wv*_1_ and *wv*_2_ color coded balls for the two treatment arms, respectively. To perform a subject randomization, a ball is randomly drawn from the urn and replaced. The subject is assigned to the treatment, e.g. 1, based on the color of the ball selected. After that, *βv*_2_ balls are added to the urn for treatment 2. Otherwise, *βv*_1_ balls are added to the urn for treatment 1 [4]. The conditional allocation probability of the mUD in this case is$p_{i,1}\left(mUD\right)=\frac{wv_{1}+n_{i-1,2}\beta v_{1}}{wv_{1}+wv_{2}+n_{i-1,1}\beta v_{2}+n_{i-1,2}\beta v_{1}}.$ Since *v*_1_+*v*_2_ = 1 and both*w* and*β* are positive real numbers, this formula can be simplify to $p_{i,1}\left(mUD\right)=\frac{v_{1}+n_{i-1,2}\beta v_{1}}{1+n_{i-1,1}\beta v_{2}+n_{i-1,2}\beta v_{1}}.$ When*β*=0, the mUD is reduced to the CR. When*β* > 0, and w.l.o.g., assuming0 < *v*_2_ < *v*_1_ < 1, the unconditional allocation probability for the second subject is:

(1) $$u_{2,1}=v_{1}\frac{v_{1}}{1+\beta v_{2}}+v_{2}\frac{v_{1}+\beta v_{1}}{1+\beta v_{1}}<v_{1}\frac{v_{1}}{1+\beta v_{2}}+v_{2}\frac{v_{1}+\beta v_{1}}{1+\beta v_{2}}=v_{1}.$$

This inequality suggests that the unconditional allocation for the second assignment is affected by the value of parameter*β*. For example, with *v*_1_:*v*_2_ = 2/3:1/3 and*β*=1, *u*_2,1_ = 3/5 = 0.6. Similar calculation leads to*u*_3,1_ = 558/845 = 0.5905. These results are consistent with Kaiser's findings [7]. When*β*=10, *u*_2,1_ = 0.4214. As*β* approaches infinity, *u*_2,1_ approaches*v*_2_ = 1 − *v*_1_.

Although it is desirable for a randomization design to have an unconditional allocation probability that equals the target allocation probability at each treatment assignment, not all randomization designs have this property [8], [10]. However, it is necessary for all randomization designs to have an unconditional allocation probability that converges to the target allocation asymptotically. Let$r_{j}=\underset{i\to \infty }{\lim}\left(n_{ij}/i\right)$ be the asymptotic allocation ratio for the mUD. When*i*→∞, the conditional allocation probability is

(2) $$p_{i1}\left(i\to \infty \right)=\underset{i\to \infty }{\lim}\frac{v_{1}+n_{i-1,2}\beta v_{1}}{1+n_{i-1,1}\beta v_{2}+n_{i-1,2}\beta v_{1}}=\frac{\left(1-r_{1}\right)v_{1}}{r_{1}v_{2}+\left(1-r_{1}\right)v_{1}}=r_{1}.$$

This leads to a quadratic equation $\left(v_{1}-v_{2}\right)r_{1}^{2}-2v_{1}r_{1}+v_{1}=0$ with its positive root representing the asymptotic allocation of the mUD as a function of the target allocation.

(3) $$r_{1}=\frac{v_{1}-\sqrt{v_{1}v_{2}}}{\left(v_{1}-v_{2}\right)}$$

Based on Eq. (3), if the original target allocation is 2:1 = 0.6667:0.3333, the mUD will asymptotically approach to the allocation0.5858:0.4142 = 2:1. Similarly, if the original target allocation is3:1 = 0.75:0.25, the asymptotic allocation will be 0.6340:0.3660 = 3:1, and so on. These results demonstrate that the mUD does not satisfy the necessary condition for a valid randomization algorithm, in two-arm unequal allocation scenario.

### 3.2 The provisional allocation

Although the mUD discussed above does not converge to the original target allocation*v*_1_:*v*_2_, as shown in Section 3.1, it does converge to an allocation*r*_1_:*r*_2_. This could be exploited to adjust the original target allocation provisionally, (hence termed provisional allocation) that would lead to the target allocation asymptotically. This goal is achieved by setting the provisional allocation in Eq. (3) to $\frac{r_{1}^{2}}{r_{2}^{2}}=\frac{{\left(v_{1}-\sqrt{v_{1}v_{2}}\right)}^{2}}{{\left(\sqrt{v_{1}v_{2}}-v_{2}\right)}^{2}}=\frac{v_{1}^{2}-2v_{1}\sqrt{v_{1}v_{2}}+v_{1}v_{2}}{v_{1}v_{2}-2v_{2}\sqrt{v_{1}v_{2}}+v_{2}^{2}}=\frac{v_{1}}{v_{2}}.$ For example, to target an unequal allocation 2:1 = 0.6667:0.3333, use a provisional allocation of2^2^:1 = 0.8:0.2. That is, using the allocation4:1 in the mUD, the randomization procedure will guarantee the allocation 2:1 in the long run, as shown in Eq. (3). Similarly, to achieve 3:1 = 0.25:0.75, or2:1 = 0.5858:0.4142, one can use the provisional allocation3^2^:1 = 0.9:0.1, or 2:1 = 0.6667:0.3333, respectively. This procedure is named as modified urn design with provisional allocation (mUD-PA).

### 3.3 The mUD-PA procedure

For a two-arm trial with a target allocation of *r*_1_:*r*_2_, the mUD-PA procedure starts from*r*_1_ and *r*_2_ color coded balls for the two treatment arms respectively. When a subject is ready for randomization, a ball is randomly drawn from the urn and replaced. The subject is assigned to the corresponding arm. If a treatment 1 ball was drawn, add$\beta r_{2}^{2}$ balls to the urn. Otherwise, add$\beta r_{1}^{2}$ balls to the urn. The conditional allocation probability for this procedure is

(4) $$p_{i,1}\left(mUD-PA\right)=\frac{r_{1}+\beta r_{1}^{2}n_{i-1,2}}{1+\beta r_{2}^{2}n_{i-1,1}+\beta r_{1}^{2}n_{i-1,2}},\;\;\left(i=1,2,\cdots \right)$$

**Fig. 1:**
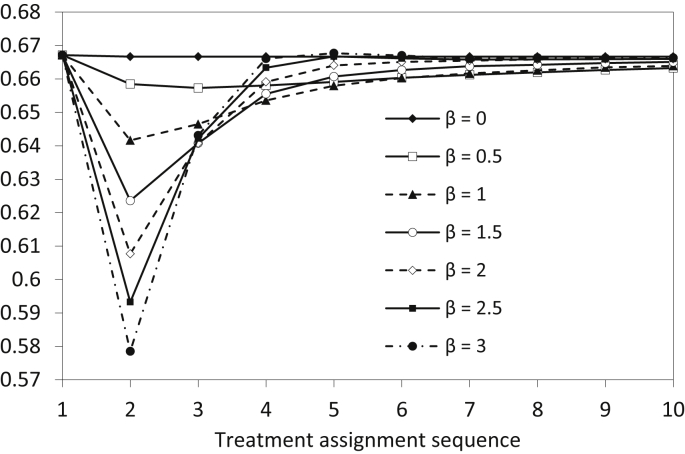
Unconditional allocation probability under mUD-PA. Target allocation 2:1, simulation = 100,000/scenario.

**Fig. 2:**
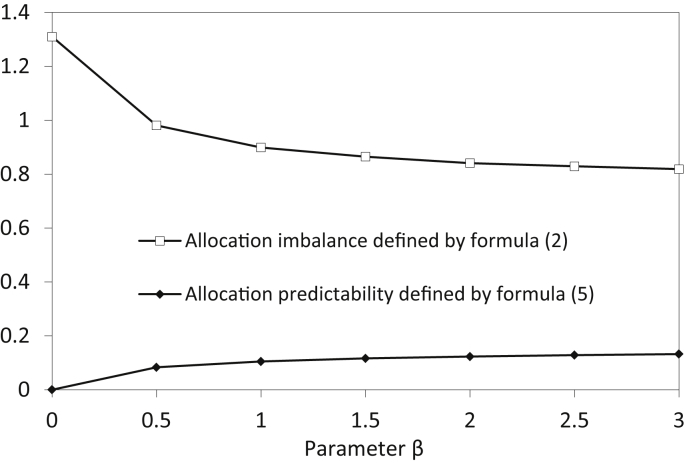
Allocation imbalance and predictability under mUD-PA. Target allocation 2:1, n = 12, simulation = 100,000/scenario.

While the asymptotic allocation is invariant to*β*, the speed of allocation convergence, the allocation imbalance, and the allocation predictability are affected by*β*. Fig. 1 shows the unconditional allocation probabilities for the first 10 treatment assignments for various values of*β*. Notice, the larger the*β*, the faster the convergence of the unconditional allocation probability is achieved. Also notice, for the second assignment, larger*β* leads to larger shift in the unconditional allocation probability from the target allocation probability (see inequality (1)). In Fig. 2, the allocation imbalance and the allocation predictability, as defined in Section 2, are shown for the target allocation of 2:1. Notice in Fig. 2 with sample size *n* = 12, smaller*β* is associated with larger allocation imbalance and lower allocation predictability. The figure seems to suggest that*β* between 1.0 and 1.5 might be desirable for the target allocation of2:1,.

Under the mUD-PA, the unconditional allocation probability has small but noticeable drifts from the target allocation for the second and third treatment assignment. It quickly converges to the target allocation after that. It is worthwhile to emphasize that the mUD-PA is capable to accurately target any desired two-arm unequal allocations, including those involving large integers, such as 13:8, as well as irrational numbers, such as2:1. This feature makes the mUD-PA applicable for complex allocations even when the sample size is small. For example, for allocation 13:8, the PBR requires a minimal block size of 21, which is useful only when the sample (or stratum) size is large. On the other hand, the mUD-PA with a provisional allocation of $\frac{13^{2}}{13^{2}+8^{2}}:\frac{8^{2}}{13^{2}+8^{2}}=0.7253:0.2747$ can achieve the desired allocation when the sample size is not large.

## 4 An urn design for unequal allocation via equal allocation

In his recent paper, Kaiser suggested that “*a solution for r*_1_:*r*_2_ *treatment allocation is to perform randomization for balanced allocation tor*_1_ + *r*_2_ *treatment arms*, *and then combiner*_1_ *of these arms for the experimental arm assignment and r*_2_ *of these arms for control* [7].” By applying this strategy to Wei's UD, a modified urn design via equal allocation (mUD-EA) is defined for targeting unequal allocations. For trial ***A*** comparing *m* ≥ 2 arms with *unequal* allocation*r*_1_:*r*_2_:⋯:*r*_*m*_, envision trial ***B*** comparing $M=\sum_{j=1}^{m}r_{j}$ arms with *equal* allocation. Let $T_{i}^{A}$ and $T_{i}^{B}$ be the treatment assignment for the *i*th subject in trials ***A*** and ***B*** respectively. The mapping relationship between$T_{i}^{A}$ and $T_{i}^{B}$ is $T_{i}^{A}=k$ when$\sum_{j}^{k-1}r_{j}<T_{i}^{B}\leq \sum_{j}^{k}r_{j}$, with*r*_0_ = 0. The mUD-EA procedure works in the same way as Wei's UD(*w,α,β*) for equal allocations, except the treatment mapping step. The conditional allocation probability for the mUD-EA procedure is:

(5) $$p_{i,j}\left(mUD-EA\right)=\frac{r_{j}+\alpha n_{i-1,j}+\beta r_{j}\left(i-1-n_{i-1,j}\right)+\beta n_{i-1,j}\left(r_{j}-1\right)}{M+\alpha \left(i-1\right)+\beta \left(M-1\right)\left(i-1\right)}$$

Here constant*α* could be positive, negative or zero, parameter*β* is a positive number. Although allocation elements*r*_*j*_ are not necessarily integers, it is required that$M=\sum_{j=1}^{m}r_{j}>1.$ As Wei's UD treats the *M* arms symmetrically, the unconditional allocation probability under the mUD-EA is preserved for each treatment assignment. The mUD-EA applies to all unequal allocations in trials with two or more arms without loss of allocation accuracy. Similar to Wei's UD, the mUD-EA behaviors close to the CR as the sample size increases.

## 5 Comparisons of statistical and operational properties via simulation studies

In the previous two sections both the mUD-PA and the mUD-EA were argued to be valid randomization algorithms for unequal allocations. Currently, the PBR and the CR are the two most commonly used methods for unequal allocations. Both of them have unconditional allocation probabilities equal to the target allocation for each treatment assignment. A few other randomization designs have also been proposed in the recent years, including the maximal procedure (MP) [13], the block urn design (BUD) [14], and the mass weighted urn design (MWUD) [8]. Using the notations specified in Section 2, the conditional allocation probability for the PBR can be obtained from an urn model [5]:

(6) $$p_{ij}\left(PBR\right)=\frac{br_{j}\left(1+k_{i-1}\right)-n_{i-1,j}}{b\left(1+k_{i-1}\right)-\left(i-1\right)}\text{,}$$

where *k*_*i*_ _−_ _1_ = *int*(*i* − 1/*b*), with*int*(*x*) denoting the largest integer not exceeding*x*. The same urn model can be used for the BUD, with the exception of the ball return rule [14]:

(7) $$p_{ij}\left(BUD\right)=\frac{r_{j}b+r_{j}k_{i-1}-n_{i-1,j}}{b+k_{i-1}-\left(i-1\right)},\;$$

$\text{where}\;\;k_{i-1}=\underset{1\leq j\leq m}{\min}(\mathrm{int}(n_{i-1,j}/\left(br_{j}\right)))\text{.}$ When the minimal block size is used, the BUD and the PBR are identical. The MWUD starts from one ball in the urn for each treatment, with the mass of the balls proportional to the target allocation probability, and the sum of the mass of all balls equals*b*. The probability a ball being picked in a random draw is proportional to its mass. After the subject is assigned to the treatment, the mass of this ball is reduced by one unit before the ball is returned to the urn. This unit amount of mass is distributed to all treatment arms based on the allocation ratio [8]. The conditional allocation probability for the MWUD is:

(8) $$p_{ij}\left(MWUD\right)=\frac{\max\left[br_{j}-n_{i-1,j}+\left(i-1\right)r_{j},0\right]}{\sum_{h=1}^{m}\max\left[br_{h}-n_{i-1,h}+\left(i-1\right)r_{h},0\right]}\;\;$$

Included in the simulation study are four trial scenarios composed by two levels for sample size (small and large) and two types of allocation elements (small integers and irrational numbers). In practice, a large sample size may exceed 1000. However, restricted randomization designs are most likely applied within strata formed by the combination of baseline covariate categories, where 100 is considered large for an average stratum size. Eight randomization designs are included for each trial scenario. The parameters for each randomization design are selected with the consideration of performance optimization. Treatment imbalance is evaluated by the average allocation accuracy, the average allocation precision, and standard deviation of the treatment arm size. Allocation randomness is evaluated by the average allocation predictability, the proportion of deterministic assignments and the proportion of complete random assignments.

**Fig. 3:**
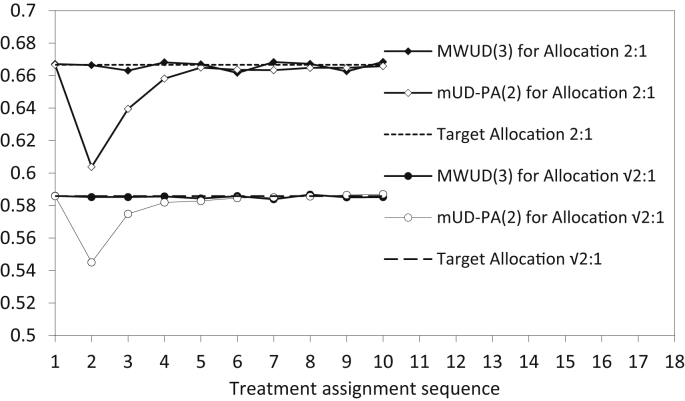
Unconditional allocation probability under MWUD & mUD-PA. Simulation = 100,000/scenario.

As shown in Table 1, for small trials with unequal allocations composed by small integers, both mUD-PA(2) and mUD-EA(0,2) could be considered as good options, if the allocation randomness is the primary concern. For allocations involving irrational numbers, both mUD-PA(2) and mUD-EA(0,2) can accurately target the desired allocation and offer low allocation predictability and treatment imbalance similar to that for PBR(12). For large trials, the allocation imbalances for both mUD-PA(2) and mUD-EA(0,2) are significantly larger than those for PBR, MUD, MP, and MWUD. This is expected because mUD-PA and mUD-EA are extensions of Wei's UD, which is designed mainly for small trials. However, when the desired allocation includes irrational numbers, the disadvantage of the PBR becomes clearer as the sample size increases. When the randomization algorithm cannot target the desired allocation, as the sample size increases, the treatment allocation accuracy will decrease proportionally. In this situation, the mUD-PA and mUD-EA remain valuable if allocation randomness is important. In general, the MWUD offers a better combination of low allocation predictability and small allocation imbalance than other randomization designs; although the unconditional allocation probabilities for the first few assignments are not exactly the same as the target allocation probability. Fig. 3 shows the unconditional allocation probability for the first 10 assignments under MWUD(3) and mUD-PA(2).

**Table 1:** Performance Comparison of Randomization Designs for Unequal Allocations 10,000 simulations per scenario

Sample Size n	Randomization Design	Allocation Ratio		Allocation Imbalance Measures			Allocation Randomness Measures			Strictly Preserves Allocation Ratio
		Desired Allocation $r_{1}^{*}:r_{2}^{*}$	Target Allocation $r_{1}:r_{2}$	Allocation Accuracy ${\bar{d}}^{*}$	Allocation Precision $\bar{d}$	Arm Size Stdev ${\hat{\sigma }}_{n_{1}}$	Allocation Predictability $\bar{\varphi }$	Deterministic Assignment $\mathrm{Pr}\left(p_{ij}=1\right)$	Complete Random $\mathrm{Pr}\left(p_{ij}=r_{ij}\right)$	
10	CR	2:1	2:1	1.216		1.496	0	0	1	Yes
	PBR(3), BUD(3)			0.440		0.473	0.283	0.401	0.400	Yes
	PBR(6)			0.603		0.597	0.201	0.200	0.320	Yes
	BUD(6)			0.687		0.593	0.164	0.097	0.280	Yes
	MWUD(3)			0.642		0.580	0.187	0.064	0.299	
	mUD-PA(2)			0.793		0.912	0.129	0	0.268	
	mUD-EA(0,3)			0.923		1.084	0.082	0	0.242	Yes
	CR	√2:1	√2:1	1.278		1.549	0	0	1	Yes
	PBR(5), BUD(5)		3:2	0.573	0.544	0^‡^	0.283	0.301	0.200	Yes
	PBR(12), BUD(12)		7:5	0.887	0.887	0.660	0.140	0.032	0.100	Yes
	MWUD(3)		√2:1	0.682		0.611	0.205	0.016	0.100	
	mUD-PA(2)		√2:1	0.829		0.951	0.141	0	0.100	
	mUD-EA(0,2)		√2:1	0.896		1.032	0.112	0	0.100	Yes
100	CR	2:1	2:1	3.562		4.691	0	0	1	Yes
	PBR(3), BUD(3)			0.421		0.472	0.311	0.440	0.340	Yes
	PBR(6)			0.559		0.594	0.246	0.280	0.272	Yes
	BUD(6)			0.697		0.595	0.187	0.119	0.206	Yes
	MWUD(3)			0.648		0.585	0.211	0.073	0.227	
	mUD-PA(2)			2.075		2.714	0.054	0	0.092	
	mUD-EA(0,2)			2.552		3.372	0.034	0	0.077	Yes
	CR	√2:1	√2:1	3.737		4.923	0	0	1	Yes
	PBR(5), BUD(5)		3:2	1.136	0.543	0^‡^	0.283	0.300	0.200	Yes
	PBR(12), BUD(12)		7:5	0.825	0.803	0.847	0.200	0.143	0.090	Yes
	MWUD(3)		√2:1	0.692		0.603	0.227	0.017	0.010	
	mUD-PA(2)		√2:1	2.203		2.842	0.058	0	0.010	
	mUD-EA(0,2)		√2:1	2.419		3.150	0.045	0	0.010	Yes

CR: Complete randomization PBR(b): Permuted block randomization (block size) BUD(b): Block urn design (block size) MWUD(b): Mass-weighted urn design (parameter b) mUD-PA(β): Modified urn design with provisional allocation mUD-EA(α,β): Modified urn design via equal allocation						Allocation accuracy: $d_{i}^{*}={\left[{\left(n_{i,1}-ir_{1}^{*}\right)}^{2}+{\left(n_{i,2}-ir_{2}^{*}\right)}^{2}\right]}^{1/2}$ Allocation precision: $d_{i}={\left[{\left(n_{i,1}-ir_{1}\right)}^{2}+{\left(n_{i,2}-ir_{2}\right)}^{2}\right]}^{1/2}$ Allocation predictability: $\varphi_{i}={\left[{\left(p_{i,1}-r_{1}\right)}^{2}+{\left(p_{i,2}-r_{2}\right)}^{2}\right]}^{1/2}$				

‡ Occurs when the sample size is a multiple of the block size.

Fig. 3 demonstrates that, the shift in the unconditional allocation probability for MWUD(3) is trivial. For the mUD-PA(2), after three assignments, the difference between the unconditional allocation probability and the target allocation probability is negligible.

## 6 Discussion

Wei's UD is well known and has been widely used for equal allocations. As unequal allocations are receiving more attention in clinical trials, the generalization of Wei's UD to unequal allocation provides investigators more options than just the CP, which may result unwanted imbalances, and the PBR, which has been criticized for its vulnerability to selection bias due to the low allocation randomness and may be unable to target the desired allocation accurately. The two randomization procedures presented in this manuscript, the mUD-PA and the mUD-EA, offer alternatives with desirable features, especially when the sample size is small and the target allocation involves large or irrational numbers. In this manuscript the presentation of the mUD-PA was limited to two-arm scenarios. The extension of the provisional allocation strategy to trials with more than two arms requires additional works. With the availability of the mUD-EA and the MWUD, both are useful for*m* ≥ 2 unequal allocations, immediate need for these extensions may not be warranted.
